# Variability of vitamins B1, B2 and minerals content in baobab (*Adansonia digitata*) leaves in East and West Africa

**DOI:** 10.1002/fsn3.184

**Published:** 2014-11-17

**Authors:** Traoré Hyacinthe, Parkouda Charles, Korbo Adama, Compaoré-Sérémé Diarra, Mamoudou H Dicko, Jan J Svejgaard, Bréhima Diawara

**Affiliations:** 1 Département Technologie Alimentaire, IRSAT/CNRST03 BP 7047, Ouagadougou 03, Burkina Faso; 2 Institut d'Economie Rurale (IER), Programme Ressources Forestières, CRRA-SotubaBP 258, Bamako, Mali; 3 Département de Biochimie- Microbiologie, Laboratoire de Biochimie Alimentaire Enzymologie Biotechnologie Industrielle et Bioinformatique (BAEBIB), UFR/SVT Université de OuagadougouOuagadougou 03 BP7021, Burkina Faso; 4 Landscape and Planning, Centre for Forest, University of Copenhagen1958, Frederiksberg, Denmark

**Keywords:** Baobab, leaves, minerals, variation, vitamins

## Abstract

The regional variability and age–age correlation on vitamin B1, vitamin B2 and minerals (Ca, Mg, P, K, Cu, Fe, Mn, Na, and Zn) concentration in baobab leaves were investigated. Baobab was cultivated from seeds from 11 countries including Benin, Burkina Faso, Kenya, Malawi, Mali, Mozambique, Niger, Tanzania, Togo, Senegal, and Sudan. Vitamins B1 and B2 content were assessed using microbiological VitaFast kits methods and minerals by atomic absorption and flame spectrometry methods. Overall, the results showed a higher content of vitamin B2 compared to vitamin B1 with the highest vitamin B2 content (1.04 ± 0.05 mg/100 g DM) from Senegal. The highest iron (Fe) content of 26.39 mg/100 g was found in baobab leaves from Mali. For age–age correlation, adult baobab leaves of Nankoun in Burkina Faso provided the highest calcium (Ca) content of 3373 mg/100 g. However, for provenance trial, young plants from three communities of Burkina Faso showed the highest calcium (Ca) and potassium (K) content. The study demonstrated that vitamins B1 and B2 and mineral contents in baobab leaves vary with the country and the age of the tree. Vitamin B1 content was higher in baobab leaves from ascendants compared to those from descendants, while in contrast vitamin B2 content was higher in the leaves from the descendants compared to their ascendants (mother tree).

## Introduction

In several regions of Africa (Sahel) as well as elsewhere, leafy vegetables play an important role in people's lives. Indeed, in these areas, leafy vegetables provide income for rural populations, in addition to being a source of essential nutrients such as vitamins and minerals, which often lack from the staple-based meals (Kahane et al. 2005). Among these leafy plants, stands baobab (*Adansonia digitata* L.) wherein its products (leaves, fruits and bark) are used for food and medicinal treatment. The leaves are mostly consumed as leafy vegetables with cereal meals in some West African countries (Sidibe and Williams 2002). The nutrients in the leaves are believed to compensate the deficiency of some nutrients such as vitamins and minerals in the cereals. Previous studies reported that baobab leaves contained (terms of Dry Matter) proteins (13–15%), carbohydrates (60–70%), fat (4–10%), fiber (11%), ash (16%), minerals (calcium, magnesium, potassium and iron), and a significant level of vitamins (A, B1, B2, and C); 80% of the energy value which ranges from 1180 to 1900 kJ/100 g is metabolizable energy (Becker 1983; Yazzier et al. 1994; Nordeide et al. 1996; Wickens and Lowe 2008). Several studies have been conducted on the distribution, taxonomy, agro-ecology and the variation of the morphology and nutrient content of baobab fruit (Yazzier et al. 1994; Nordeide et al. 1996; Collière 2002; Sidibe and Williams 2002; Diop et al. 2005; Parkouda et al. 2012). However, there are few data on the variation of the biochemical composition of baobab leaves, and much less on their age–age correlation (mother trees compared to progenies). However, it is known that the composition of tree products can be influenced by a combination of genetic and environment effects (Nour et al. 1980; Zheng et al. 2009a,b). Indeed, investigation on the physical characteristics and nutrient content of baobab fruit from 11 populations of baobab showed a high variability in all measured parameters, within and between populations (Parkouda et al. 2012).

The existence of significant variation of the content of vitamins B1 and B2 and minerals may indicate a possibility for a potential selection and domestication of superior tree for production of seeds to be used in vegetable gardens. The production of leave with high nutrient content from the superior tree can then contribute to alleviate the most important forms of malnutrition in most African countries (deficiencies in micronutrients such as vitamins, iron, and zinc). Consequently, the aim of the present study was to investigate both regional variation and age–age correlation of the biochemical composition of baobab leaves, including vitamins B1, B2, and minerals (Ca, Cu, Fe, Mg, Mn, K, Na, P, and Zn).

## Material and Methods

### Areas of study

**Figure 1:**
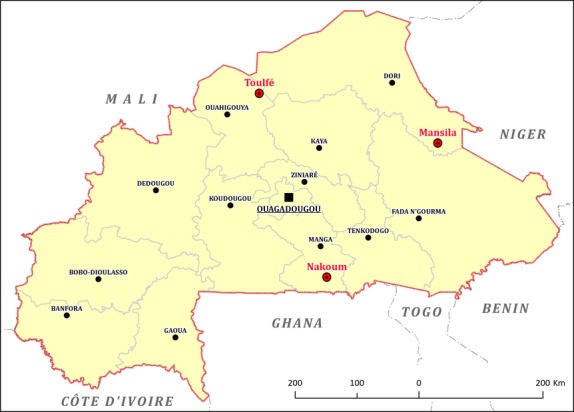
Provenance of baobab samples in Burkina Faso for age-age correlation test.

**Figure 2:**
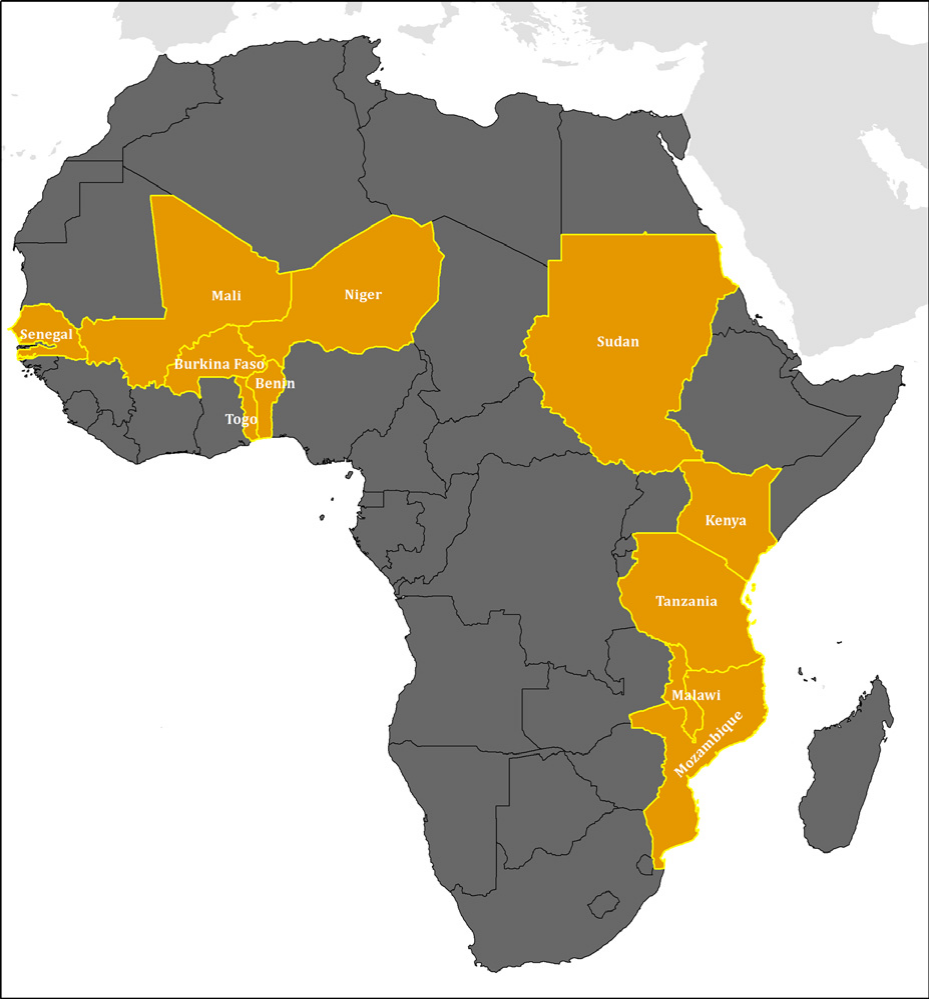
Provenance of baobab seeds samples used for the provenance test.

Baobab leaf samples (204) were collected in Burkina Faso and Mali. In Burkina Faso, sampling involved three localities in three different phytogeographical zones, namely: Mansila in Yagha Province, Nankoun in Nahouri Province, and Toulfé in Loroum Province (Fig.1). Samples from Mali used for provenance test included leaves of young plants grown from baobab seeds, which originated from 11 African countries namely: Benin, Burkina Faso, Kenya, Malawi, Mali, Mozambique, Niger, Senegal, Sudan, Tanzania, and Togo (Fig.2).

### The provenance tests

Provenance test was realized at the Agricultural Research Station/Sotuba, Institute of Rural Economy of Mali (12.63°N and 7.91°W). It included seeds from 30 communities (Table1) from 11 East and West African countries (Benin, Burkina Faso, Kenya, Malawi, Mali, Mozambique, Niger, Tanzania, Togo, Senegal, and Sudan). Geographical and climatic data from provenances and experimental plan are detailed in Korbo et al. (2012).

**Table 1:** Baobab seeds collection sites for the provenance trial

	Site	Country
1	Boukoumbé	Benin
2	Dassa	
3	Peni	Burkina Faso
4	Kolangal	
5	Nobéré	
6	Kibwezi	Kenya
7	Rumphi	Malawi
8	Mangochi	
9	Kourougué	Mali
10	Samé	
11	Komodiguili	
12	Koumadiobo	
13	Zambougou	
14	Nabougou	
15	Manica	Mozambique
16	Torodi	Niger
17	Parc w	
18	Maradi	
19	Bandia	Senegal
20	Kordofan sud	Soudan
21	Kordofan nord	
22	Kordofan ouest	
23	Dodoma	Tanzanie
24	Iringa	
25	Kilimandiaro	
26	Morogoro urbain	
27	Rivage	
28	Morogoro rural	
29	Togo 1	Togo
30	Lama	

### The age-age correlation tests

The age–age correlation test was performed on 44 adult baobab trees selected in three localities of three phytogeographical areas in Burkina Faso. The seeds of baobab were harvested and sown at the research station in Sotuba. The experimental plan was a randomized complete block with three repetitions. Each repetition included 30 seeds of each 44 adult baobabs.

### Preparation of samples

Baobab leaves were harvested with a part of their stems, packed into food bags, put immediately in a cooler containing ice for transportation to the laboratory, and stored in a −20°C freezer. The leaves used to perform the provenance test were harvested from 14-month-old (after sowing) seedlings. For the age–age correlation test, leaves were at first, directly collected from 44 adult baobabs, and secondly collected from the 2-month-old seedlings after sowing.

To determine vitamin B1 and B2 content, 5 g of fresh leaves of each sample, petiole, and main veins were removed and collected. They were ground with 10 mL of distilled water and using a grinder (Waring Blendor, New Hartford, Connecticut, USA). The resulting homogenate was bottled in containers of 100 mL, and stored in a freezer at −20°C until needed for analysis. To determine mineral content, fresh leaves were dried in the laboratory at room temperature (25°C) and then ground using a porcelain mortar. Five grams of powder from each sample were collected in bags and sent to BUNASOLS (Ouagadougou, Burkina Faso) for analysis. Three samples were collected from each of the baobab leaves (adult and young plants) and analysis run in duplicate.

### Chemical analysis

Water content was determined according to the NF V 03-707: 2000 (AFNOR, 2000). The content is obtained by difference weighing of 5 g of the sample before and after oven-drying (modell 600; Memmert, Schwabach, Germany) at 105 ± 1°C for 12 h. Vitamins B1 and B2 content were determined by a microbiological reference method using VitaFast kits (Art. No. P1006 and P1007, R-Biopharm AG, Darmstadt, Germany) according to the manufacturer's instructions (Institute FürProduktqualität GmbH). Briefly, 1 g of the sample was weighed and transferred into a 50 mL sterile centrifuge vial. Twenty milliliter of deionized water was added, shaken and the pH adjusted to 4.5 with HCl. For vitamin B1 determination; 300 mg of taka diastase is added whereas for vitamin B2, 300 mg of taka diastase, and 10 mg of acid phosphatase were added. The mixture was well shaken and incubated for 1 h in the darkness at 37°C. After incubation, 40 mL deionized water was added and the extract was heated for 30 min in a water bath at 95°C (during extraction the vials were well shaken five times). The extract was then quickly chilled down to below 30°C. The clear supernatant was diluted in 1.5 mL sterile vials with sterile water from the test kit. For the next steps, only sterile samples, which were diluted with sterile water from the test kit were pipetted onto the microtiter plate. The plate was incubated at 37°C in the darkness for 48 h. After incubation, the turbidity was measured with microtiter plate reader at 630 nm and the content of vitamin B1 and vitamin B2 determined. The mineral elements were determined in the dried leaves of baobab according to the method described by Houba et al. (1980). Briefly, after Kjeldahl mineralization with sulfuric acid in the presence of salicylic acid and selenium, phosphorus was assayed by an auto analyzer (1000; SKALAR, Erkelenz, Germany); calcium and magnesium were determined by atomic absorption (100; Perkin Elmer, Hamburg, Germany); potassium and sodium were determined using a flame photometer (400; Corning, New York, USA). The other minerals such as iron, copper, manganese, and zinc were determined by atomic absorption after acid digestion in the presence of nitric acid (30%), sulfuric acid (96%), and perchloric acid (70%).

### Statistical analysis

For vitamins B1 and B2 provenance trial, data were computed in Excel and analyzed. Statistical analysis was focused on analysis of variance (ANOVA). These analysis were performed with a risk of error *P* = 5%. The Fisher test was used to compare the differences among means. The comparative study of the minerals between samples was performed by SAS software (Version 6 Edition 1985, SAS Institute Inc., Cary, NC). PRINTCOMP software and CORR are used to test the correlation between minerals of baobabs ancestors and their descendants. The correlation method used was the Pearson product moment correlation coefficient.

## Results and Discussion

### Vitamins B1and B2 content related to provenance

The content of vitamins B1 and B2 from provenance trials is reported in Table2. Baobab leaves from Malawi had the highest content of vitamin B1 (0.41 mg/100 g) whilst those from Mozambique, Tanzania, and Senegal had the lowest content (0.16 mg/100 g). For vitamin B2, the highest content of 1.04 mg/100 g was found in baobab leaves from Senegal and the lowest (0.20 mg/100 g) recorded in baobab leaves from Mozambique. Compared to vitamin B1, vitamin B2 content is higher for all samples except those from Niger and Togo. Significant difference was found in vitamin B1 and vitamin B2 content between countries in general (*F* values were, respectively, 2.39 and 4.85). Difference on vitamin B1 and B2 level according to provenance has also been reported by Maranz et al. (2008). As reported elsewhere, the difference can be explained mostly by germplasm differences, since the leave samples included in the present study were taken from trees grown in the same field under the same environmental conditions and were analysed using uniform procedures (Maranz et al. 2007).

**Table 2:** Levels of vitamin B1 and B2 according to the origin (mg/100 g DM)

Country	Vitamin B1	Vitamin B2
Benin	0.39 ± 0.24	ND
Burkina Faso	0.31 ± 0.15	0.63 ± 0.32
Kenya	0.18 ± 0.06	0.58 ± 0.27
Malawi	0.41 ± 0.00	0.47 ± 0.22
Mali	0.23 ± 0.09	0.67 ± 0.30
Mozambique	0.16 ± 0.02	0.20 ± 0.01
Niger	0.25 ± 0.08	0.22 ± 0.01
Tanzania	0.16 ± 0.02	0.85 ± 0.34
Togo	0.27 ± 0.06	0.24 ± 0.05
Senegal	0.16 ± 0.06	1.04 ± 0.05
Sudan	0.37 ± 0.14	0.47 ± 0.20
Average	0.26 ± 0.10	0.54 ± 0.27
F value	2.39^1^	4.85^1^

ND, not determined.

1 Significant (For each parameter (Vitamin B1, Vitamin B2), at least one country has an average significantly different from the other countries at 95% confidence).

### Vitamins B1 and B2 content related to age–age correlation

The results of vitamin B1 and B2 from age–age correlation tests are reported in Table3. The highest average value of vitamin B1 (0.28 mg/100 g) was found in adult baobab leaves from Mansila and the lowest (0.15 mg/100 g) in leaves of young plants from Pô. The leaves of adult baobabs from Pô had the highest vitamin B2 content (0.93 mg/100 g), whereas their young plants have the lowest content (0.14 mg/100 g). However, content of vitamin B1 were higher in the leaves of adult baobabs compared with their young plants and contrarily for vitamin B2, which is of higher amount in the leaves of young baobabs.

**Table 3:** Levels of vitamins B1 and B2: age-age correlation tests (mg/100 g DM)

	Vitamin B1		Vitamin B2	
Origin	Old	Young	Old	Young
Mansila	0.28 ± 0.23	0.22 ± 0.06	0.24 ± 0.39	0.51 ± 0.44
Pô	0.18 ± 0.07	0.15 ± 0.02	0.14 ± 0.04	0.93 ± 0.01
Toulfé	0.21 ± 0.10	0.16 ± 0.03	0.27 ± 0.30	0.50 ± 0.44
Average	0.22 ± 0.05	0.18 ± 0.04	0.22 ± 0.07	0.65 ± 0.25
*P* value	0.0556^1^	0.7187NS	0.0067^2^	0.12NS

NS, not significant.

1 Significant.

2 Very significant.

Vitamins B1 and B2 content in baobab leaves were determined to highlight the variability related to provenance origins, and the age–age correlation. A significant difference in vitamin B2 content between adult baobabs (*P* = 0.0067) was reported in the present study. The average levels of vitamins B1 and B2 from the test of provenance and the test of age–age correlation are similar to those obtained in baobab leaves from the Bla locality (0.33 and 0.77 mg/100 g) and the Cinzana locality (0.41 and 0.86 mg/100 g) in Mali (Maranz et al. 2008). The average vitamin B1 content in this study was higher than those in baobab from Senegal (0.13 mg/100 g) (Kerharo 1980). The high vitamin B2 content compared to vitamin B1 was also observed in baobab leaves from Mali and Senegal (Kerharo 1980; Maranz et al. 2008). Indeed, in the previous study, Vialard-Goudou et al. (1958) had noticed that green leafy vegetables are good sources of vitamin B2.The variation is probably a combination of genetic and environmental effects as reported by Nour et al. (1980) and Zheng et al. (2009a,b). Indeed, the composition of products can be influenced significantly by the environment such as soil type, fertilizer, water, or sunlight intensity as reviewed by Chadare et al. (2009). In the present study, the difference can be explained specifically by the genetic effects since all baobabs were grown in the same habitat. Studies on other species revealed moderate to high heritability for vitamin C and sugar content; for instance high vitamin C heritability have been shown in Kiwi (*Actinidia deliciosa*) and *Capsium anuum* (Cheng et al. 2004; Gelata and Labuschagne 2006); in sugar beet (*Beta vulgaris*) high sugar content heritability under various environments has also been demonstrated (Ober et al. 2004).

### Minerals content related to provenance

The results of the minerals content for provenance tests are summarized in Table4. The data showed a very heterogeneous distribution of these minerals among provenances although leaves were harvested at the same experimental station, and therefore were subject to similar soil composition, climate, and environmental conditions. The disparity is much higher for phosphorus, copper, sodium, and zinc. Baobab leaves from Mozambique showed the highest values of calcium (2458 mg/100 g) and sodium (2.43 mg/100 g); Tanzania held the highest potassium content (2677 mg/100 g) while Mali and Niger, respectively, had the highest concentrations in iron (26.39 mg/100 g) and zinc (17.83 mg/100 g). Leaves of the Baobab tree (*A. digitata* L.) are widely consumed in semi-arid Africa as a leafy green vegetable (Boffa 1999; NRC 2006, 2008). In previous studies, *A. digitata* leaves have been indeed reported to be a very rich in calcium (Smith et al. 1996; Glew et al. 1997). Statistical analysis showed a significant difference between the mean values of copper, sodium, zinc, and phosphorus between provenance (*P* = 0.0001). Those of calcium, magnesium, manganese, iron, and potassium also presented significant differences between provenances with *P*-value of 0.0008, 0.0088, 0.0059, 0.0011, and 0.0004, respectively.

**Table 4:** Content of minerals in the leaves according to the origin (mg/100 g DM)

Origins	Ca	Mg	P	K	Cu	Fe	Mn	Na	Zn
Benin	1771 ± 135	685 ± 68	434 ± 94	1226 ± 64	0.51 ± 0.22	15.39 ± 0.79	10.13 ± ± 1.18	1.20 ± 0.09	8.02 ± 2.31
Burkina Faso	2168 ± 419	887 ± 172	337 ± 58	2049 ± 513	0.61 ± 0.18	16.94 ± 8.21	11.94 ± 3.69	1.39 ± 0.27	16.70 ± 7.78
Kenya	1801 ± 360	941 ± 201	457 ± 20	1739 ± 114	0.36 ± 0.16	11.60 ± 1.87	10.11 ± 1.78	1.60 ± 0.07	6.58 ± 0.28
Malawi	2201 ± 336	814 ± 136	333 ± 53	1923 ± 624	0.83 ± 0.22	18.60 ± 6.60	11.20 ± 5.09	1.80 ± 0.41	13.60 ± 8.83
Mali	2252 ± 415	802 ± 219	351 ± 117	1792 ± 462	0.79 ± 0.30	26.39 ± 22.66	12.99 ± 3.54	1.48 ± 0.39	14.87 ± 9.81
Mozambique	2458 ± 288	771 ± 51	478 ± 191	2013 ± 375	0.84 ± 0.32	22.68 ± 15.93	10.21 ± 3.16	2.43 ± 0.43	6.56 ± 3.35
Niger	2215 ± 429	777 ± 233	267 ± 93	1552 ± 551	0.71 ± 0.50	16.91 ± 6.64	11.93 ± 4.27	1.19 ± 0.25	17.83 ± 9.70
Tanzania	1961 ± 360	814 ± 225	372 ± 139	2677 ± 439	0.76 ± 0.28	23.28 ± 7.79	9.37 ± 1.79	1.37 ± 0.29	16.05 ± 11.65
Togo	1820 ± 401	616 ± 78	283 ± 104	1295 ± 32	0.85 ± 0.06	14.04 ± 1.59	16.16 ± 5.83	1.19 ± 0.16	8.41 ± 3.46
Senegal	2366 ± 520	985 ± 92	444 ± 51	1497 ± 227	0.45 ± 0.30	23.34 ± 10.41	15.48 ± 4.24	1.22 ± 0.19	17.71 ± 16.75
Sudan	1923 ± 394	757 ± 119	550 ± 55	1588 ± 236	1.42 ± 0.50	14.60 ± 1.89	10.39 ± 5.25	1.37 ± 0.25	11.12 ± 1.98
*P* value	0.0008	0.0088	<0.0001	0.0004	<0.0001	0.0011	0.0059	<0.0001	<0.0001

### Minerals content related to age–age correlation

**Figure 3:**
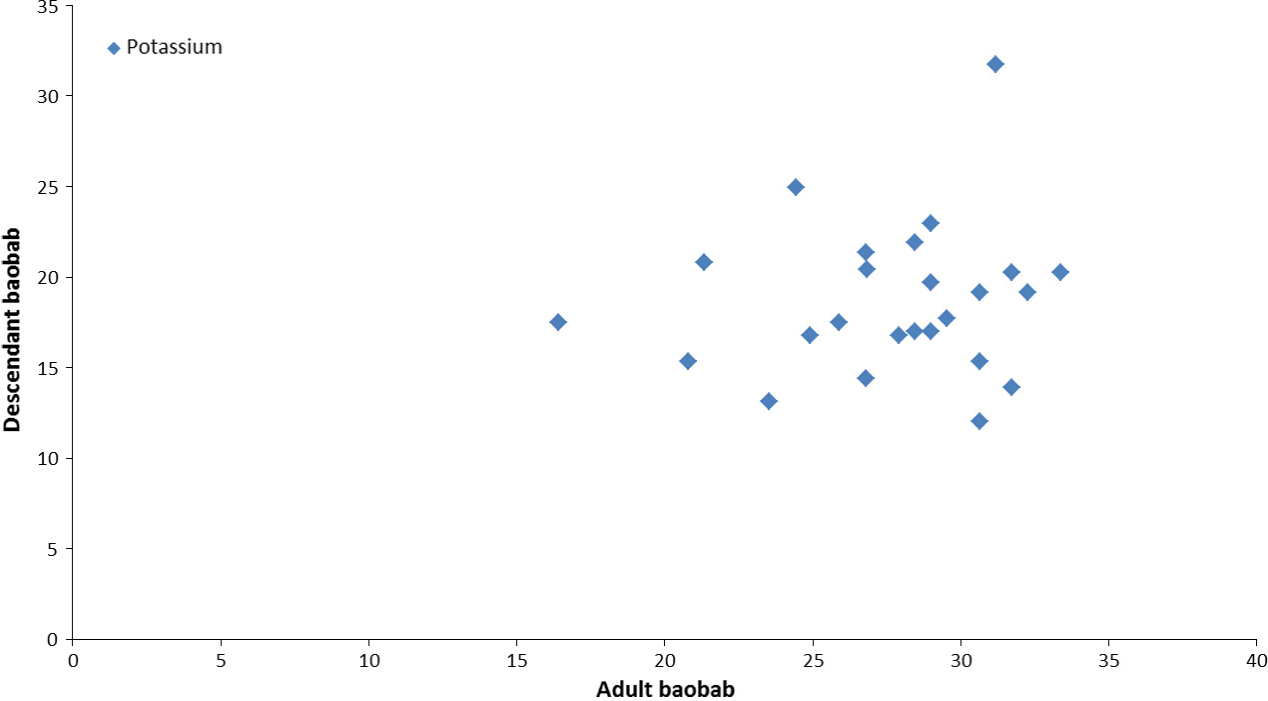
Relation between potassium content in the adult baobabs and their descendants.

**Figure 4:**
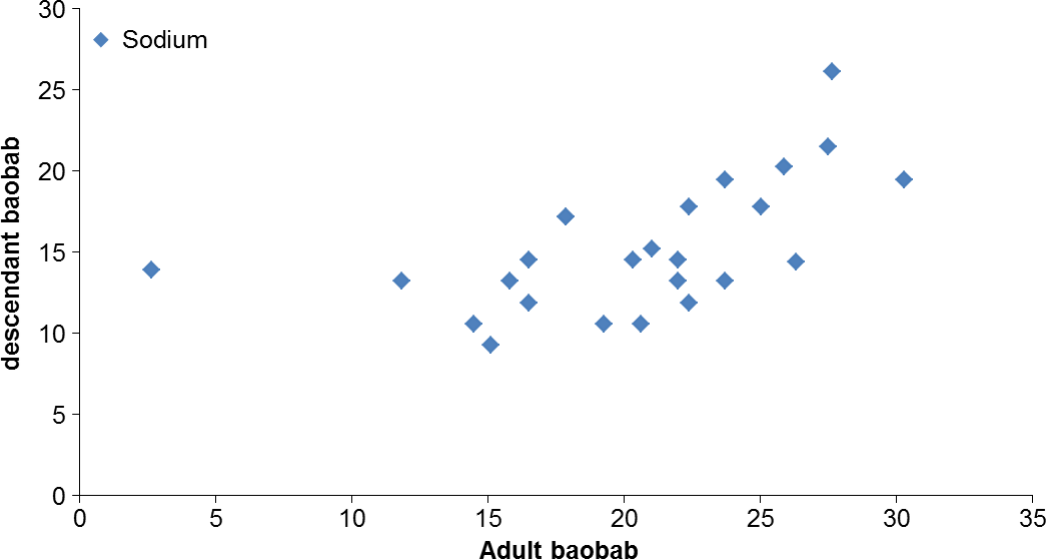
Relation between Sodium content in the adult baobabs and their descendants.

Results of minerals content for the age–age correlation tests are summarized in Table5. From the data recorded, mineral content are higher in leaves of young baobabs compared to adults with the exception for Nankoun locality with regard to calcium and copper. Young baobabs from Toulfé yielded the highest content of calcium (2140 mg/100 g), magnesium (704 mg/100 g), potassium (2809 mg/100 g), iron (27.22 mg/100 g), manganese (9.27 mg/100 g), and zinc (19.50 mg/100 g). The mother tree of Nankoun had the highest calcium content (3373 mg/100 g) but also the lowest zinc content (5.19 mg/100 g). Statistical analysis showed a significant difference only for calcium (*P* = 0.0002). The correlation between mineral content of adult baobabs (mother tree) and those of their descendants was positive only for sodium and potassium (Figs3, 4). The results showed a very heterogeneous distribution of mineral contents between provenances, although the leaves were cultivated and harvested on the same site. This heterogeneity is much greater for phosphorus, copper, sodium, and zinc. The major minerals were calcium, potassium, magnesium, and phosphorus. Iron and zinc contents are also important, however, copper and sodium contents were the lowest. Minerals such as calcium, potassium, magnesium, phosphorus, and zinc where found in higher quantities compared to those previously recorded in Mali (Maranz et al. 2008): calcium (1235 mg/100 g), potassium (1512 mg/100 g). These results are also higher compared to those recorded in Burkina Faso (Glew et al. 1997), Nigeria (Yazzier et al. 1994; Nordeide et al. 1996), and Senegal (Kerharo 1980). The iron content is slightly lower (28 mg/100 g) compared to figures from Mali (Maranz et al. 2008). However, it should be noted that the calcium and iron content in baobab leaves are higher than those observed in the leaves of *Cassia tora* (608 mg/100 g and 6 mg/100 g), *Hibiscus sabdariffa* (214 mg/100 g and 4.9 mg/100 g); *Manihot esculenta* (303 mg/100 g and 7.6 mg/100 g), *Leptadenia* sp (398 mg/100 g and 4.8 mg/100 g), and *Ficus gnaphalocarpa* (74 mg/100 g and 15.8 mg/100 g) (Toury et al. 1963). From the present study, it is shown that calcium and potassium content are very high in leaves of provenance tests (14 months old) and in leaves of young baobabs (2 months old) from the three localities in Burkina Faso. The variation observed in the Baobab leaves with regard to minerals content may have been due to either a different genetic of the plants or probably due to environmental effects and variation in minerals content might be attributed to the environmental and geological conditions in the regions (Ibrahim et al. 1974). On other hand, the difference can be explained by the fact that minerals play an important role in the plant by accumulating carbohydrates, maintaining cell turgor and plant resistance to frost, drought, and diseases (http://www.ecosociosystemes.fr/nutrition_plantes.html). It could also be said that age affects the mineral content in the leaves of baobab.

**Table 5:** Content of minerals in the leaves according to the age (mg/100 g DM)

Origin	Mansila	P mansila	Nankoun	P nankoun	Toulfé	P toulfé
Calcium	1954 ± 325	2063 ± 205	3373 ± 763	2057 ± 237	1704 ± 703	2140 ± 297
Magnesium	508 ± 93	493 ± 66	490 ± 138	563 ± 83	506 ± 199	704 ± 149
Phosphorus	242 ± 74	577 ± 53	241 ± 63	578 ± 98	207 ± 35	560 ± 145
Potassium	1991 ± 371	2523 ± 117	1751 ± 389	2714 ± 325	1935 ± 470	2809 ± 487
Cupper	0.34 ± 0.26	0.54 ± 0.07	1.05 ± 0.23	0.69 ± 0.19	0.87 ± 0.32	0.82 ± 0.27
Iron	10.32 ± 1.84	19.31 ± 7.67	9.77 ± 2.51	27.18 ± 10.27	10.17 ± 5.56	27.22 ± 10.71
Manganese	7.01 ± 2.68	7.29 ± 1.29	2.55 ± 0.72	8.96 ± 1.66	8.13 ± 3.15	9.27 ± 1.92
Sodium	1.77 ± 0.37	2.31 ± 0.25	1.32 ± 0.29	1.82 ± 0.20	1.57 ± 0.44	2.06 ± 0.58
Zinc	9.80 ± 9.50	13.83 ± 11.26	5.19 ± 2.82	8.43 ± 4.90	7.75 ± 10.43	19.50 ± 1.36

P, young plants.

## Conclusion

The present study showed that Baobab leaves had a large nutritional potential. Based on the minerals, Vitamin B1 and B2 content, Baobab has a potential to improve nutrition for millions of people in Africa. Indeed, utilization of the edible leaves as vegetables is important as reported elsewhere (NRC 2006, 2008; Parkouda et al. 2012). Therefore, baobab leaves could be used in food fortification to alleviate malnutrition problems related to micronutrients such as iron and zinc deficiencies. However, in order to properly manage the potential of Baobab leaves, basic knowledge about the bioavailability of the mineral is still lacking. The baobab leaves quality and safety could be affected by the postharvest processing and storage indicating that it is necessary to study the effect of post-harvest processing on the leaves quality and safety in order to establish good manufacturer practices for producers.
